# Supplementary material for: Barriers to the Digitization of Health Information: A Qualitative and Quantitative Study in Kenya and Lao PDR Using a Cloud-Based Maternal and Child Registration System
Source: Int J Environ Res Public Health. 2021 Jun 8;18(12):6196. doi: 10.3390/ijerph18126196 (PMC8228682; doi:10.3390/ijerph18126196)
Supplement: Supplementary file 1 [file ijerph-18-06196-s001.zip › Supplement S1.pdf]

Supplement document1: WIRE system description

Pregnant Women and Infants Registration (WIRE) Digital System

System overview

WIRE system

The WIRE system is a cloud-based registration system of mothers and children developed by Nagasaki University and TLS Co. Ltd (Nagasaki, Japan). It is designed to be used in medical institutions and has been developed to expect medical institution staff to record the information in the MCH handbook electronically. The system has two profiles; mother's and children's profiles. The former records data at ANC visits, preventative services, and delivery reports. The latter includes data relating to child welfare clinics (CWC) visits, growth monitoring, and vaccination history.

For the WIRE-Laos system, we localized our pre-existing English version to the Lao language and adjusted the MCH variables to match the Lao Mother & Child Health Book, which was also modeled after the same Japanese Mother & Child Health book (Boshi Techo).

Hardware

This project utilizes a Dell Latitude 3570 Notebook PC with Intel® Core™ i5-6200U CPUs (Dell Technologies Inc., Round Rock, TX, USA) with Windows 10 64-bit operation. (Figure 1C) The PC accesses a web-based WIRE software system through Internet Explorer, where pregnant women and infants can be enrolled, and their respective ANC or CWC data be inputted. In addition, the generated code from the digital system is attached to the Mother & Child Health book as a unique identifier. The code is based on the 14 characters result from the system.

Database

The patient's medical data was uploaded to and downloaded from a Structured Query Language (SQL) database on a Windows server at Nagasaki University-Institute of Tropical Medicine in Nagasaki, Japan, over 3G networks from Safaricom Ltd.(Nairobi, Kenya) in Kenya and Lao Telecom (Vientiane, Laos) or Unitel (Vientiane, Laos) in Lao PDR. The aggregated patient records can be exported by Excel or CSV data format for data analysis. Patient's personal and medical information are separated to ensure privacy, with only a unique WIRE system-specific ID number shared (Figure S1). The access to the WIRE system is controlled by usernames and passwords, which are encrypted by a hashing algorithm.

System users and profiles

User types

The system has three types of users: Medical professionals, Health Care Workers (HCWs), and Investigators. Depending on their assigned group, the user control different functions in the system. The user access and tasks are available in the table below:

Users' category	Access level	Tasks
Medical professionals at Clinics	Patient profiles (edit/review).	1. New patient registration.<br>2. Updating patient follow up sheet.

		3. Reviewing registered patient history.<br>4. View site-specific summary
Health Care Workers (HCWs)	Patient profiles (edit/review)	1. New patient registration.<br>2. Updating patient follow up sheet.<br>3. Reviewing registered patient history.
Investigators	Patient profiles (review only)	1. Reviewing registered patient history.<br>2. Variable adjustment<br>3. View System-wide and site-specific summary

Each user profile will include a user name, password, user type, and assigned health facility.

<u>Profiles</u>

Mother and Infant profiles are linked together for ease of access.

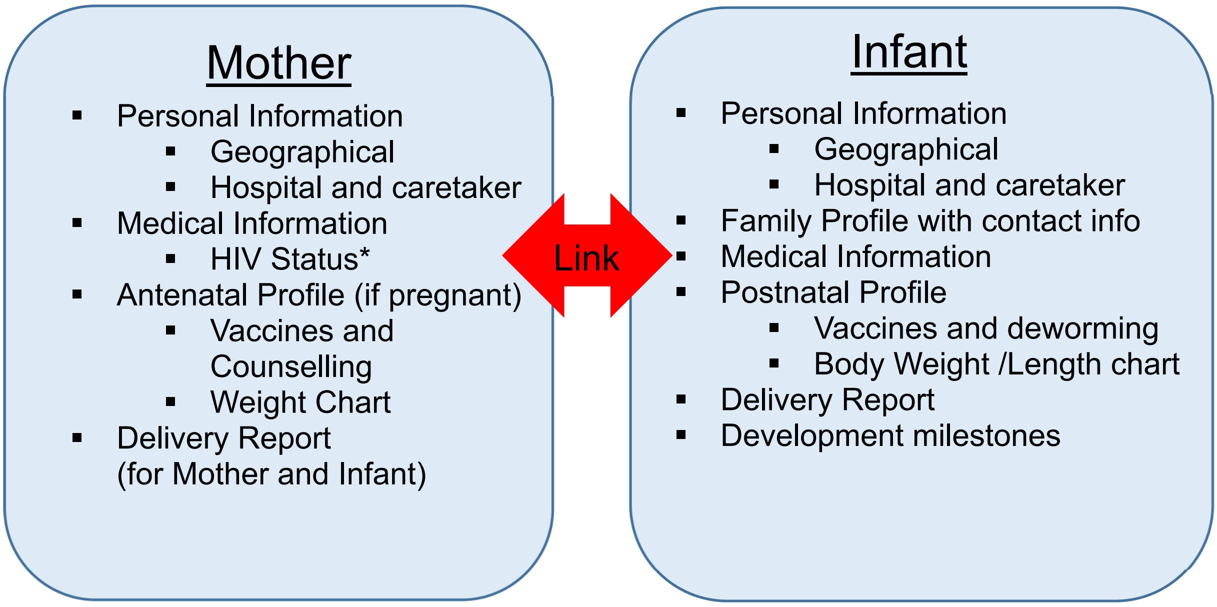

Data structure

Modular Design

The WIRE system implements a modular data design that allows flexibility for future development. Mother and Infant profiles have basic personal and geographic information and HIV status. An additional module relating to the birth of infants is included, which is accessible from either Mother or Infant profiles. In future versions, more detailed medical history, including ANC reports, PNC reports, and vaccination history, will be added. The figure below is an overview of the current and future overall data structure.

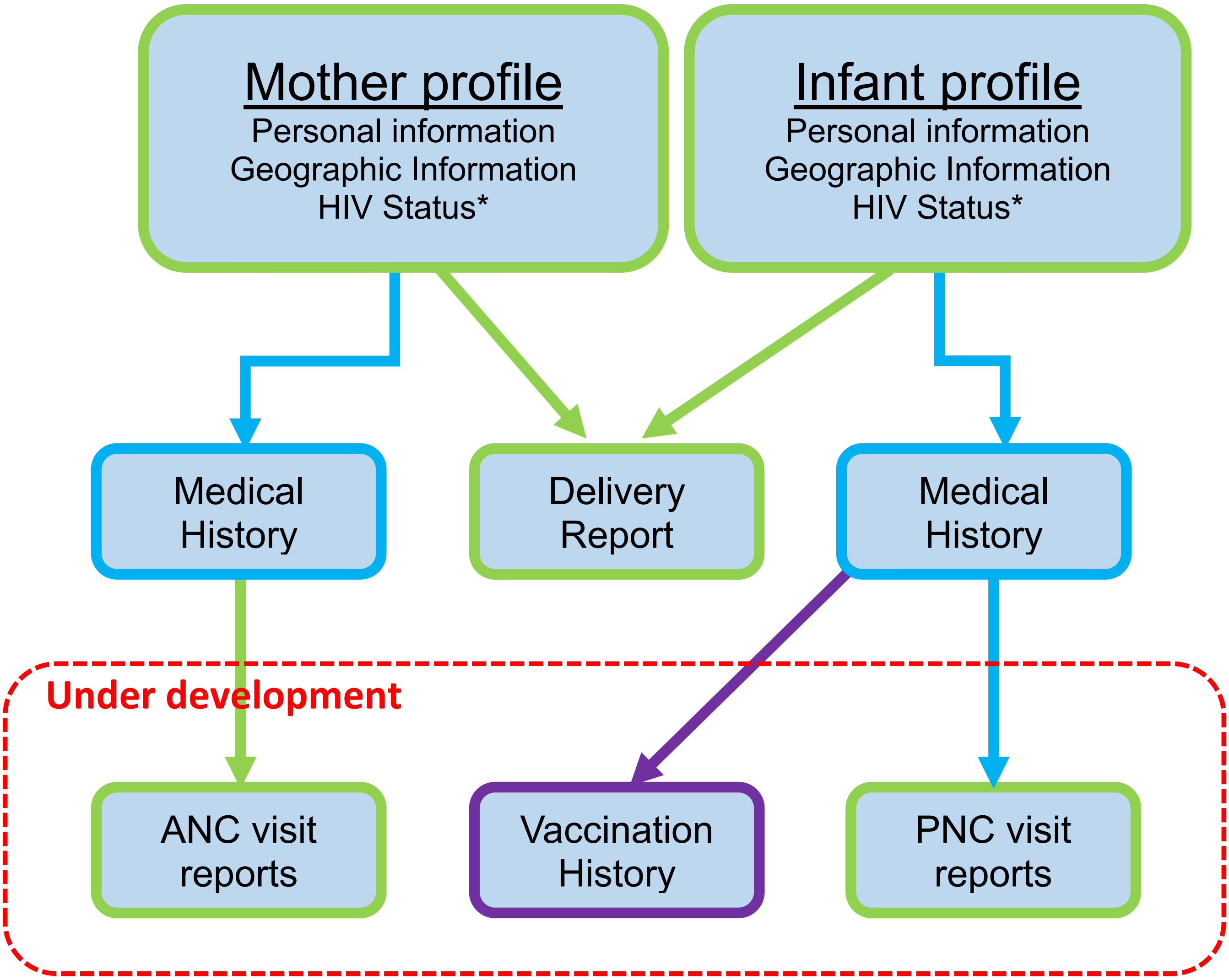

Summary and Reporting

Exporting data

The system exports three types of excel files that describe either individual patient information, medical records, or follow-up visit information. The "patients.xlsx" information includes Patient name, ID (National ID or Birth notification number), Birth certificate number, Date of birth, Age, Address, Phone number, Administrative region, and Health facility. The "medical_records.xlsx" includes basic medical information but only uses the patient's National ID or Birth notification number as an identifier. The "follow-up.xlsx" demonstrates visit by visit record of procedures and results done over time. Again, patients are only be identified by their ID.

Supplement document1: WIRE system description

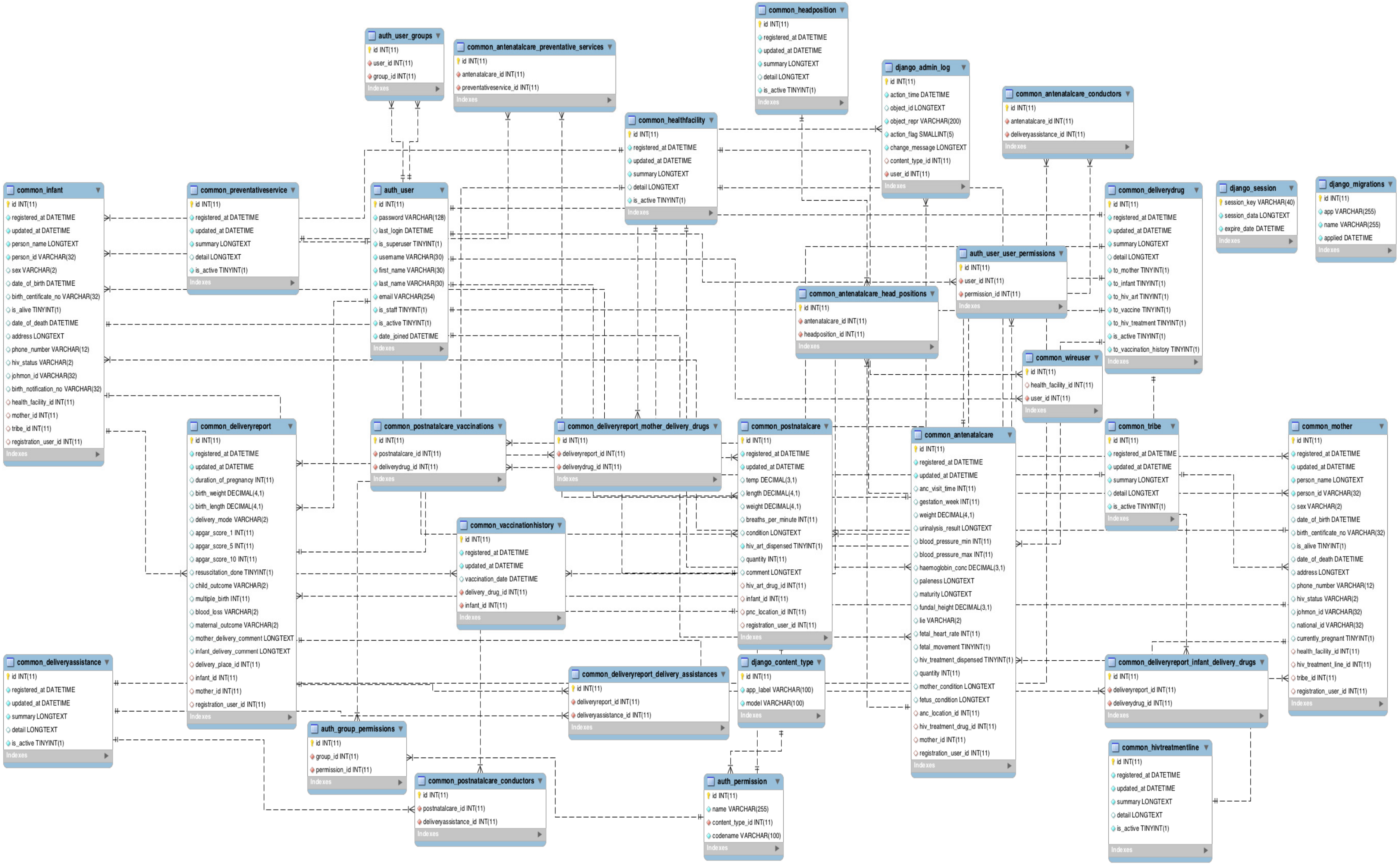

Figure S1: *Database schema for Mother and Child digital system*
