# Supplementary material for: Barriers to the Digitization of Health Information: A Qualitative and Quantitative Study in Kenya and Lao PDR Using a Cloud-Based Maternal and Child Registration System
Source: Int J Environ Res Public Health. 2021 Jun 8;18(12):6196. doi: 10.3390/ijerph18126196 (PMC8228682; doi:10.3390/ijerph18126196)
Supplement: Supplementary file 1 [file ijerph-18-06196-s001.zip › Supplement S2a.pdf]

WIRE Survey Protocol for mothers and health facility staff survey (Lao PDR)

Introduction:

The main objective of this study was to evaluate the feasibility of a sustainable and centralized electronic nationwide mother and child health digital system by focusing on registering pregnant women, mothers, and infants in Lao PDR.

Nagasaki University with TLS Co. Ltd (Nagasaki, Japan) has developed a software system called WIRE (**W**omen and **I**nfant **RE**gistration system) that enrolls and tracks pregnant women and infants during antenatal care visits (ANC) and infants postnatal care (PNC) at health facilities. WIRE is a digital records system for Lao maternity and child health clinics based on the currently used Mother & Child Health Booklet.

As of August 2017, the WIRE system has been placed in two sites in Xepon District and two sites in Songkhone District, Lao PDR. In October 2018, another site was added in Xepon District (total two sites). WIRE is operated by Maternal and Child Health (MCH) nurses (2-3 per site).

Site locations:

A) Xepon District:
    1. Xepon District Hospital
    2. Dongsavah Health Center
    3. Manchy Health Center

B) Songkhon District:
    4. Songkhon District Hospital
    5. Lahanam Health Center

Objective and Targets:

To be successful, the WIRE system must integrate into the daily workflow of MCH clinics and assist the nurses in delivering care to their patients
Determine the perception and concerns from healthcare workers using the WIRE system

1. Determine the perception and concerns from MCH nurses to identify possible barriers
2. Identify WIRE system performance and new requirements for Lao MCH settings

At each survey site, we will interview WIRE system operator (MCH nurses)

Method:

We will conduct in-depth interviews with the MCH nurses from all five sites. The interview questions are available on the following pages of this document.
Personnel and Language: These interviews will be conducted by Angkhana Lasaphonh in the Lao Language. Then the interviewer will prepare an English transcript for the interview.

User-name: ___________________________________ Interviewer: ______________________

Date: ______________________ Location :______________________

WIRE Satisfaction Survey for Health Facility Staff (Lao PDR)

Introduction: Who they are and what position do they have?

Have they used computer systems before? Yes or No

If yes: What type of system was it?

Describe the normal workflow?

(When do you provide service and register mothers)

How did the computer system integrate into and affect the Hospital/Health Centre/Dispensary environment?

Example: this is just for explanation, the more details the better interview

They are both familiar with computer systems since they have used them before.

Type of system used: *Two Desktop in data feeding – HIV/Aids for CCC clients and The desktops had specific software installed for HIV case management.*

A. Description of workflow
   - *i) When patients arrive they wait for us at …………………….*
   - *ii) ANC seeking patients go to the examination room*
   - *iii) Mothers then proceed with their children to immunization rooms so that the child can get the correct immunization dose. After immunization, we fill the registration.*
   - *iv)* 1st registration is done in paper registries then 2nd step is data to be entered in the WIRE system

1. Was the system easy to use for you and the mothers?
   Yes or No Why?:

   Follow-up Questions:

   - Time to finish one registration (minutes)
   - How long did it take to become comfortable with the system?
   - Did you try another software before that you find easy? If yes, what was it?
2. Were mothers open to enrolling themselves and their children into the WIRE system?

   Yes or No

   If Yes: What concerns did they have about the computer system?

User-name: ___________________________________ Interviewer: ______________________
Date: _______________________ Location :______________________

How did you overcome the concerns?

If not, why? What was the reason for refusal or hesitancy? If you tried to explain to them, Why do you think they did not accept after that? Could you give examples of reasons for refusing?

3. Did you perceive any benefits in your work from the system? Yes and No
   What benefits did you see from the WIRE system to your everyday work?

   How did using the computer system affect your workload? Do you prefer to make all working paper or computerized? And why?

4. What difficulties (not related to WIRE but related to work environment) did you encounter when using the system? What technical issues did you encounter?

   Expected responses: Time, not enough machines, lack of consent forms, union strikes,

   Internet network connection, power outages, unable to find names, page performance, variables not matching (missing variables, Parts missing)

   How did you solve these issues?

5. Did you find any benefits from the system to the current maternal and child health system?

   Yes or No

   Do you know how Hospital/Health Centre/Dispensary can utilize WIRE and mother's or infant's information to improve your current maternal and child health program?

6. What new features would you like to have in future versions of the system?

   What kind of functions that you find useless and time-consuming?

7. What comments or recommendations do you have for future versions of the system?

8. In your opinion, What do you think of the reasons for low coverage of WIRE data concerning paper registries in Ante Natal Care (ANC), Deliveries, Post Natal Care (PNC)?
   Please refer to the attached coverage excel sheets for each facility in the e-mail and provide the coverage percentage to the nurse for October, November, and December 2018.
