# Supplementary material for: Barriers to the Digitization of Health Information: A Qualitative and Quantitative Study in Kenya and Lao PDR Using a Cloud-Based Maternal and Child Registration System
Source: Int J Environ Res Public Health. 2021 Jun 8;18(12):6196. doi: 10.3390/ijerph18126196 (PMC8228682; doi:10.3390/ijerph18126196)
Supplement: Supplementary file 1 [file ijerph-18-06196-s001.zip › Supplement S2b.pdf]

WIRE Survey Protocol for mothers and health facility staff survey (Kenya)

Introduction:

The main objective of this study was to evaluate the feasibility of a sustainable and centralized electronic nationwide mother and child health digital system by focusing on registering pregnant women, mothers, and infants in Kwale County, Kenya.

Nagasaki University with TLS Co. Ltd (Nagasaki, Japan) has developed a software system called WIRE (**W**omen and **I**nfant **RE**gistration system) that enrolls and tracks pregnant women and infants during antenatal care visits (ANC) and infants postnatal care (PNC) at health facilities. WIRE is a digital records system for Lao maternity and child health clinics based on the currently used Mother & Child Health Booklet.

As of March 2017, the WIRE system has been placed in six sites in Kwale County, Kenya, and is operated by either maternal and child health clinic nurses, facility medical records officers, or community health workers.

Site locations:

- Kwale District Hospital
- Kinango Hospital (ANC and PNC departments)
- Diani Health Clinic
- Mwaluphamba Dispensary
- Mwachinga Dispensary
- Vyongwani Dispensary
-

Objective and Targets:

To be successful, the WIRE system must integrate into the daily workflow of MCH clinics and assist the nurses in delivering care to their patients
Determine the perception and concerns from healthcare workers using the WIRE system

1. Determine the perception and concerns from MCH nurses to identify possible barriers
2. Identify WIRE system performance and new requirements for Lao MCH settings

At each survey site, we will interview:

1. WIRE system operator
   (MCH nurses, community health workers, and medical records officer)
2. Clinical officer

Method:

We will conduct in-depth interviews with the MCH nurses, facility medical records officers, community health workers, and clinical officers from all six sites. The interview questions are available on the following pages of this document.

User-type: Nurse   Records Officer   Clinical Officer   Mother   Interviewer: _______________________
Date: _______________________   Location :_______________________

Personnel and Language: These interviews will be conducted by Daniel Harrell and Yvonne Muthiani in the local language. Then the interviewer will prepare an English transcript for the interview.

User-type: Nurse Records Officer Clinical Officer Mother Interviewer: ______________________
Date: ______________________ Location :______________________

WIRE Satisfaction Survey for Health Facility Staff

Introduction: Who they are and what position do they have?

Have they used computer systems before? Yes or No

If yes: What type of system was it?

Describe the normal workflow?

(When do you register mothers)

How did the computer system integrate into and affect the Hospital/Health Centre/Dispensary environment?

1. Was the system easy to use for you and the mothers?

   Yes or No

   Why?:

   Follow-up Questions:

   - Time to finish one registration (minutes)
   - How long did it take to become comfortable with the system?

2. Were mothers open to enrolling themselves and their children into the WIRE system?

   Yes or No

   If Yes: What concerns did they have about the computer system?
   How did you overcome the concerns?

   If not, why? What was the reason for refusal or hesitancy?

3. Did you see any increase in antenatal care visits, deliveries, and postnatal care visits compared to the previous paper-based Mother& Child book? Was there an increase in repeated ANC visits? (Skip)

4. Did you perceive any benefits from the system? Yes and No
   What benefits did you see from the WIRE system to your everyday work?

   How did using the computer system affect your workload?

User-type: Nurse Records Officer Clinical Officer Mother Interviewer: ______________________
Date: ______________________ Location :______________________

5. What difficulties (environmental) did you encounter when using the system? What technical issues did you encounter?

   Expected responses: Time, Not referred by nurses, not enough machines, lack of consent forms, union strikes,

   Internet network connection, power outages, biometric searches, unable to find names, page performance, variables not matching MCB (missing variables, Parts missing)

   How did you solve these issues?

6. Did you perceive any benefits from the system to the current maternal and child health system?

   Yes or No

   Do you know how Hospital/Health Centre/Dispensary can utilize the mother's or infant's information to improve your current maternal and child health program?

7. What new features would you like to have in future versions of the system?

8. What comments or recommendations do you have for future versions of the system?
