# Supplementary material for: Barriers to the Digitization of Health Information: A Qualitative and Quantitative Study in Kenya and Lao PDR Using a Cloud-Based Maternal and Child Registration System
Source: Int J Environ Res Public Health. 2021 Jun 8;18(12):6196. doi: 10.3390/ijerph18126196 (PMC8228682; doi:10.3390/ijerph18126196)
Supplement: Supplementary file 1 [file ijerph-18-06196-s001.zip › Supplement table S1 and S2.pdf]

Table S1. Cumulative numbers of mothers and infants, HIV status, and registered numbers of the health facilities in Kenya and Laos PDR.

**Kenya**<br>January 28th, 2017 ~ January 31st, 2020					**Lao PDR**<br>August 3rd, 2017 ~ January 31st, 2020				
	**Mothers**		**Infants**			**Mothers**		**Infants**	
Total registered	12,887		10,499		Total registered	7,132		3,528	
Mean age ± SD *(1)	26	± 6	6	± 9	Mean age ± SD *(1)	26	± 5	4	± 11
	**N**	**%**	**N**	**%**		**N**	**%**	**N**	**%**
Number of Mothers under 18y old*	412	3.2	NA	NA	Number of Mothers under 18y old*	593	8.31	NA	NA
Pregnant women*	4,382	34	NA	NA	Pregnant women*	5,867	79.6	NA	NA
Male population	NA	NA	5,307	51.1	Male population	NA	NA	1,797	51.4
**HIV status**									
	**Mothers**		**Infants**			**Mothers**		**Infants**	
**HIV test result**	**N**	**%**	**N**	**%**	**HIV test result**	**N**	**%**	**N**	**%**
reactive	342	2.7	29	0.3	reactive	7	0.1	12	0.3
non-reactive	9,601	74.5	2,579	24.6	non-reactive	3,132	43.9	1,305	37
not-tested	326	2.5	5,749	54.8	not-tested	2,704	37.9	1,962	55.6
no response	2,618	20.3	2,136	20.3	no response	1,289	18.1	249	7.1
**Health facilities distribution**									
	**Mothers**		**Infants**			**Mothers**		**Infants**	
Name of Health facility	**N**	**%**	**N**	**%**	Name of Health facility	**N**	**%**	**N**	**%**
Kinango Hospital	3,832	29.7	3,046	29	Songkhone District Hospital	3,629	50.9	1,669	47.3
Diani Health Centre	4,234	32.8	3,630	34.6	Lahanam Health Center	551	7.7	505	14.3
Kwale District Hospital	2,368	18.4	1,856	17.7	Xepon District Hospital	2,153	30.2	926	26.3
Mwaluphamba Dispensary	1,092	8.5	687	6.5	Dongsavanh Health Center	550	7.7	322	9.1
Mwachinga Dispensary	718	5.6	647	6.2	Manchy Health Center	249	3.5	106	3
Vyongwani Dispensary	524	4.1	485	4.6	No Response	0	0	0	0
Outreach	58	0.4	116	1.1					
No Response	61	0.5	26	0.3					

[1] * at the time of registration in WIRE system; (1) Age is expressed in years for mothers and in months for infants

Table S2. Cumulative visits of ANC, PNC, and registered services in the WIRE system in Kenya and Lao PDR.

	Kenya		Lao PDR			Kenya		Lao PDR	
ANC visits					PNC visits				
Total registered	6,875		12,382		Total registered	26,624		4,355	
Mean gestation week ±SD*	25	± 6	21	± 7	Mean infant age ±SD*	9	± 11	3	± 6
	N	%	N	%		N	%	N	%
First ANC visit	2,843	41.4	4,257	34.4	First PNC visits	10,041	37.7	2,549	68.5
Two ANC visit	1,707	24.8	2,523	20.4	PNC revisits	16,583	62.3	1,806	41.5
Three ANC visit	1,154	16.8	1,931	15.6					
Four ANC visits or more	1,171	17	3,671	29.6					
ANC services					PNC services				
ANC service type	N		N		PNC service type	N		N	
Breast examination	1,299				BCG vaccine (6)	2,958		1,314	
Deworming treatment	1,709				IPV (7)	1,436		366	
Feeding counselling	1,411				Measles (HIV related)	2,094		417	
ITN (1)	1,225		129		OPV (8)	8,998		1,560	
Iron and Folate	5,855		7,244		Rota virus vaccine	4,198			
IPT (2)	5,968				Pneumococcal vaccine	6,248		1,165	
TT (3)	1,644				Yellow fever vaccine	5			
Vitamin A (4)	10				Deworming treatment (9)	1,314		458	
DT (5)			3,258		Vitamin A	2,621		918	
HIV counseling			1,936		ITN	1,887		4	
HIV testing			1,934		Weight monitoring	26,487		4,355	
					Height monitoring	23,354		4,327	
					Hepatitis B vaccine			2,892	
					Penta vaccine			1,658	
					JEV (10)			241	

* age in weeks at the time of first registered visit in WIRE system, (1) Insecticide-Treated Nets, (2) Malaria prophylaxis, (3) Tetanus Toxoid, (4) 200,000 IU, (5) Diphtheria-Tetanus Toxoid, (6) Bacillus Calmette–Guerin vaccine, (7) Inactivated Polio Vaccine, (8) Oral Polio Vaccine, (9) (Albendazole 200mg/400mg), (10) Japanese Encephalitis Vaccine
